# Lactone Component From *Ligusticum chuanxiong* Alleviates Myocardial Ischemia Injury Through Inhibiting Autophagy

**DOI:** 10.3389/fphar.2018.00301

**Published:** 2018-03-29

**Authors:** Gang Wang, Guoliang Dai, Jie Song, Maomao Zhu, Ying Liu, Xuefeng Hou, Zhongcheng Ke, Yuanli Zhou, Huihui Qiu, Fujing Wang, Nan Jiang, Xiaobin Jia, Liang Feng

**Affiliations:** ^1^College of Traditional Chinese Medicine, China Pharmaceutical University, Nanjing, China; ^2^College of Pharmacy, Anhui University of Chinese Medicine, Hefei, China; ^3^Key Laboratory of New Drug Delivery System of Chinese Materia Medica, Jiangsu Provincial Academy of Chinese Medicine, Nanjing, China; ^4^Department of Clinical Pharmacology, Affiliated Hospital of Nanjing University of Chinese Medicine, Nanjing, China; ^5^Nanjing Institute of Product Quality Inspection, Nanjing, China

**Keywords:** lactone, myocardial ischemia injury, autophagy, cardiomyocyte, cardioprotection

## Abstract

The dysregulation of autophagy is associated with a series of cardiovascular diseases, such as myocardial ischemia injury. Lactone component from *Ligusticum chuanxiong* (LLC) is the major constituent of the traditional Chinese herb *L. chuanxiong* Hort., which has been reported to hold potential cardioprotective effects. In this study, to determine whether LLC protects the heart through regulation of autophagy, we explored the effects of LLC on cardioprotection and autophagy in myocardial ischemia injured rats and H9c2 cardiomyocytes. Our results showed that LLC significantly reduced infarct size and serum levels of lactate dehydrogenase, creatine kinase, and cardiac troponin and ameliorated histological features in a dose-dependent manner. Similar protections were observed in cardiomyocytes subjected to oxygen-glucose deprivation (OGD). Meanwhile, LLC inhibited autophagy induced by myocardial ischemia injury, characterized by increased autophagic vacuoles, LC3-II/LC3-I ratio and the expression of Beclin 1, whereas decreased the expression of p62. Additionally, LLC combined with a lysosomal inhibitor chloroquine (CQ) reduced LC3-II/LC3-I ratio in cardiomyocytes compared with CQ alone. Furthermore, LLC-afforded cardioprotection was abolished by a specific PI3K inhibitor LY294002. Collectively, these findings demonstrated that cardioprotective effects of LLC were related to restoration of autophagic flux through the activation of PI3K/Akt/mTOR signaling pathway.

## Introduction

Myocardial ischemia injury refers to the decrease of myocardial blood supply or the increase of myocardial oxygen demand, which exceeds the maximum blood supply, resulting in damages and changes in myocardial metabolism, function and structure. Cardiovascular disease is a common and frequently occurring disease that seriously threatens human health, and myocardial ischemia occupies a cardinal place in the cause of cardiovascular diseases (Aronow, 2006; Yang et al., 2013). Therefore, it is especially important to find effective natural products against myocardial ischemia injury.

*Ligusticum chuanxiong*, the dried root of *L. chuanxiong* Hort. (Umbelliferae), is one of the most popular used traditional Chinese medicinal herbs for treating various cardiovascular and cerebrovascular diseases such as cerebral ischemia, coronary heart disease and stenocardia. Especially, the beneficial effects of *L. chuanxiong* and its extracts on myocardial ischemia have been extensively confirmed (Jiang et al., 2014; Liu et al., 2016). A number of previous studies have demonstrated that the extracts of *L. chuanxiong* have extensive pharmacological actions of vasodilation, antiplatelet aggregation, angiogenesis, anti-inflammatory, relieving spasm and scavenging oxygen free radicals (Hou et al., 2004; Meng et al., 2008; Kim and Rhyu, 2010; Or et al., 2011). Lactone component from *L. chuanxiong* (LLC) is the most abundant and bioactive ingredient of *Ligusticum chuanxiong*, which mainly contained Z-ligustilide, senkyunolide A, senkyunolide H, and senkyunolide I. Recent studies have showed that LLC possesses therapeutic effects of anti-atherosclerotic, vasorelaxation and anti-inflammatory (Liu et al., 2005; Chan et al., 2007; Xiao et al., 2014). However, it is still unknown whether it has comparable therapeutic efficacy for myocardial ischemia injury.

Autophagy is an evolutionarily conserved intracellular lysosomal degradative process to eliminate damaged, modified or aging proteins and organelles, and which is a dynamic process involving in autophagosome formation and degradation. It is not only a widespread normal physiological process to maintain cellular homoeostasis, but also associated with pathological process of multiple diseases including cancers, neurodegenerative diseases, tissue fibrosis, and cardiovascular diseases (Lavandero et al., 2013; Ghavami et al., 2015; Menzies et al., 2017; Yoshida, 2017). Abundant evidence suggests that autophagy may be activated in the myocardium under acute or chronic ischemic conditions, but the role of autophagy in myocardial ischemia injury is still controversial (Decker and Wildenthal, 1980; Przyklenk et al., 2012; Huang et al., 2015). Hence, whether and how LLC regulates the autophagy during myocardial ischemia need to be investigated and analyzed.

To address above issues, our present study was designed to (i) characterize the concentration-dependent effects of LLC on the myocardial ischemia injury *in vivo* and *in vitro*; (ii) clarify the effects of LLC on the autophagy during myocardial ischemia; and (iii) illuminate whether the PI3K/Akt/mTOR signaling pathway is involved in potential molecular mechanism of LLC-afforded cardioprotection. The results may provide new insights into the mechanism of LLC on cardioprotection and new inspirations for the prevention and treatment of myocardial ischemia injury.

## Materials and Methods

### Materials and Reagents

*Ligusticum chuanxiong* was purchased in the pharmacy of Jiangsu Provincial Hospital of Integrated Traditional Chinese and Western Medicine (Nanjing, China). The habitat of *L. chuanxiong* Hort. is Chengdu of Sichuan Province, China, it was identified by Prof. Dekang Wu from Nanjing University of Chinese Medicine (Nanjing, China).

Dulbecco’s modified Eagle’s medium (DMEM), trypsin and 3-(4,5-dimethyl-2-thiazolyl)-2,5-diphenyl-2-H-tetrazolium bromide (MTT) were purchased from KeyGEN Biotech Co., Ltd. (Nanjing, China). Fetal bovine serum (FBS) was provided from Gibco/BRL Co., Ltd. (Grand Island, NY, United States). Dimethyl sulfoxide (DMSO), chloroquine, LY294002 and isoproterenol (ISO) were obtained from Sigma-Aldrich (St. Louis, MO, United States). Diltiazem (Dil) was obtained from Tianbian Pharmaceutical Co., Ltd. (Tianjin, China). Antibodies for LC3, PI3K and p-Akt were purchased from Cell Signaling Technology (Danvers, MA, United States), and antibodies for Beclin 1, p62, mTOR and β-actin were purchased from Boster Biological Technology Co., Ltd. (Wuhan, China), Antibody for p-mTOR was purchased from Abcam (Cambridge, United Kingdom), and antibody for Akt was purchased from Absin (Shanghai, China). Anti-rabbit secondary antibody and anti-mouse secondary antibody were purchased from Abcam (Cambridge, United Kingdom). The western blot detection reagent ECL was purchased from Beyotime Biotech Co., Ltd. (Shanghai, China). All other chemicals and reagents were purchased from local agencies.

### Preparation and Analysis of LLC

The dried roots of *L. chuanxiong* (300 g) were refluxed twice with 3000 mL of 80% (v/v) ethanol (EtOH) for 1.5 h, and all the extracts were vacuum evaporated to remove EtOH at 50°C. Subsequently, the crude extracts were loaded onto a macroporous resin D101 column. After reaching the adsorption balance, the loaded sample was eluted with 5 BV (bed volume) of different concentrations of EtOH (v/v, 0, 30, and 75%) at the flow rate of 3 BV/h. Orderly, the fraction of 75% EtOH elution was collected for rotatory evaporation at 50°C. Finally, the yield of obtained LLC was 3.43%.

Analysis of LLC was performed on an Agilent 1200 series HPLC instrument (Agilent Technologies, Waldbronn, Germany) equipped with quaternary pump, column oven and autosampler. The separation was carried out on Agilent reverse phase TC-C18 column (5 μm, 250 mm × 4.6 mm). The mobile phase gradient conditions consisted of acetonitrile (A) and 0.1% formic acid (B):0–15 min, A:5–10%; 15–16 min, A:10–15%; 16–30 min, A:15–25%; 30–40 min, A:25–25%; 40–50 min, A:25–65%; 50–65 min, A:65–91%. The flow rate was 0.8 mL/min, and the column temperature was maintained at 25°C. Detection wavelength of 300 nm was selected as the preferred wavelength for the analysis of LLC. Standard substance including senkyunolide H, senkyunolide I, senkyunolide A, and Z-ligustilide were precisely weighed to prepare a mixed solution. Subsequently, samples were injected and analyzed in a volume of 20 μL, after being passed through a 0.45 μm organic microfiltration membrane.

### Animals and Induction of Myocardial Ischemia Injury by Isoproterenol

Male Sprague-Dawley rats (200 ± 20 g) were provided by Shanghai SLAC Laboratory Animal Center (Shanghai, China) and housed in constant conditions at a temperature of 25 ± 1°C, relative humidity of 45 ± 5%, and on a 12 h light-dark cycle with access to food and water *ad libitum*. All protocols for experiment were approved by the Institutional Animal Care and Ethics Committee of Jiangsu Provincial Academy of Chinese Medicine. Isoproterenol (ISO) is a strong β-adrenergic agonist, which can increase myocardial oxygen consumption through speeding up heart rate and enhancing myocardial contractility, resulting in cardiac overload, myocardial microcirculation disturbance, coronary artery spasm, myocardial infarction, myocardial necrosis. As it is widely accepted that Isoproterenol (ISO) injection can readily induce acute myocardial ischemia injury in rats (Shi et al., 2013; Guo et al., 2016; Dhivya et al., 2017).

After a week of acclimatization, all rats were randomly divided into six groups (*n* = 10/group) named Control (Con), ISO, Dil (diltiazem, positive control drug), low-dose LLC, middle-dose LLC and high-dose LLC Groups. Low-dose LLC, middle-dose LLC and high-dose LLC Groups were orally given 20, 40, and 80 mg⋅kg^-1^ of LLC (dissolved in 0.5% w/v CMC-Na), respectively. Dil group was orally given 20 mg⋅kg^-1^ of diltiazem. Con and ISO groups were orally given 0.5% w/v CMC-Na (sodium carboxymethyl cellulose). After 14 consecutive days of pretreatment, all rats (except for control group) were subcutaneously injected with ISO at a dosage of 40 mg⋅kg^-1^ for 2 consecutive days to established acute myocardial ischemia model (Karthick and Stanely, 2006; Song et al., 2016). On the last day of experiment, the animals were sacrificed and then blood and hearts were collected for further studies.

### Myocardial Infarct Size Measurement

The myocardial infarct size was measured by 2,3,5-triphenyltetrazolium chloride (TTC, Sigma-Aldrich Co., St. Louis, MO, United States) staining as previously described (Yao et al., 2015). Briefly, the heart was frozen at -80°C for 5 min and cut into approximately 2-mm-thick slices. The slices were incubated in 1% (w/v) TTC at 37°C for 20 min in the dark, and subsequently fixed in 10% formaldehyde. Myocardial infarct size was calculated using Image-pro plus 6.0 software (Media Cybernetics, Rockville, MD, United States), and reported as the percent of infarct divided by the total area at risk.

### Analysis of Histopathology

The heart tissues were qualitatively analyzed for histological alterations by Hematoxylin-Eosin (H&E) treatment. The tissues were fixed in 10% formaldehyde and embedded in paraffin, sectioned at 5 μm, stained with H&E using standard methods. Images of staining were observed under the light microscopy (Olympus IX51, Tokyo, Japan).

### Measurements of Serum LDH, CK, and cTnI Levels

After rats being sacrificed, the serum was separated from the rat blood by centrifugation. Activities of CK and LDH in serum were determined by standard commercial kits (Jiancheng Bioengineering Institute, Nanjing, China). Serum level of cardiac troponin I (cTnI) was measured by using a rat cTnI ELISA kit (Jiancheng Bioengineering Institute, Nanjing, China) according to the manufacturer’s instructions.

### Transmission Electron Microscopy (TEM)

Rat hearts were rapidly harvested for detecting myocardial ultrastructural alterations by TEM. Briefly, a small piece (1 mm^3^) of left ventricular was fixed in a 2.5% glutaraldehyde solution, post-fixed in 1% osmium tetroxide, and dehydrated with a graded series of EtOH. Then, the sample was substituted with propylene oxide solution and embedded in Poly/Bed mixture. Ultra-thin sections were acquired by standard procedures, the thickness of ultra-thin slice was about 50–80 nm. The sections were stained with uranyl acetate and lead citrate and then observed using a transmission electron microscope (H-7650, Hitachi Limited, Japan). Quantitative morphometric analysis of autophagic vacuoles was performed by a blinded observer. Five rats were assigned in each group, and 10 fields were examined for each rat. TEM analysis was carried out as described (Zhang et al., 2014).

### H9c2 Cell Culture and Oxygen-Glucose Deprivation (OGD) Establishment

H9c2 embryonic rat cardiac cells were purchased from the Cell Resource Center of Chinese Academy of Sciences (Shanghai, China). The cells were cultured in DMEM high-glucose medium and supplemented with 10% FBS at 37°C under a humidified atmosphere containing 5% CO_2_ and 95% air.

H9c2 cells were challenged by OGD to imitate myocardial ischemia injury *in vitro* as previously described (Wang J. et al., 2015). For the OGD experiments, after cell confluency reached 80–90%, the cell culture medium was changed to serum-free and glucose-free DMEM, and then the cells were incubated in hypoxia chamber saturated with 95% N_2_ and 5% CO_2_ at 37°C for 4 h. The cells in the control group were cultured in high-glucose with 10% FBS DMEM. LLC was dissolved in 0.1% (v/v) dimethylsulfoxide (DMSO) and diluted with DMEM high-glucose medium to the concentrations of 10, 20, 40, and 80 μg⋅mL^-1^, whereas chloroquine (CQ) and LY294002 were diluted to 5 and 20 μmol⋅L^-1^, respectively. All the interventions included LLC, CQ, and LY294002 were applied 12 h prior to the onset of OGD.

### Cell Viability Assays and Measurement of LDH Release in Culture Supernatants

H9c2 cell viability was determined using MTT assay according to the manufacturer’s instructions (Wang B. et al., 2015). After OGD treatment, MTT solution was added to the wells and further incubated for 4 h at 37°C, then solubilized with 100 μL DMSO. The absorbance was measured at 490 nm using a microplate reader (Thermo, New York, NY, United States). Cell viability was calculated as follows: Viability (%) = (OD of Assay – OD of Blank)/(OD of control -OD of blank) × 100%.

To evaluate the degree of cell injury induced by OGD, LDH released into culture media were measured by LDH activity assay kit (Jiancheng Bioengineering Institute, Nanjing, China) according to the manufacturer’s instructions.

### Flow Cytometry

Cell apoptosis ratio was detected by the Annexin V-FITC/PI apoptosis assay kit (KeyGEN, Nanjing, China) according to the manufacturer’s instructions. Briefly, after attaching cells were harvested, cells were suspended in binding buffer, stained with Annexin V-FITC and PI, and early (Annexin V+/PI-) and late (Annexin V+/PI+) apoptotic cells were sorted by fluorescence-activated cell sorting (FACS) (Becton-Dickinson, United States).

### Evaluation of Fluorescent LC3 Puncta

Autophagic flux was evaluated by mRFP-GFP-LC3 assay as described previously (Ling et al., 2016). In brief, H9c2 cells were grown in 6-well plates and transfected with a mRFP-GFP-LC3 adenovirus (Genechem, Shanghai, China) at 15 multiplicity of infection (MOI) for 24 h. Then the cells were washed with PBS, fixed with 4% paraformaldehyde for 15 min, and viewed under a fluorescence microscope. Due to GFP is instable in acidic conditions of the lysosome, whereas mRFP is relatively stable, so colocalization of both red and green fluorescence indicates autophagosome induction. In contrast, red fluorescence that does not overlay green fluorescence and appears red punctuate in merged images indicates autolysosome formation. The number of dots per cell was counted to evaluate autophagic flux.

### Western Blot Analysis

The samples were obtained from rat hearts and H9c2 cells, and total protein concentrations were determined via a Bradford method. Equal amounts of protein were separated by SDS-PAGE and transferred to PVDF membranes. After blocking with 5% non-fat milk for 1 h, PVDF membranes were incubated with primary antibodies overnight at 4°C, and followed by incubation with HRP-conjugated secondary antibodies for 2 h at room temperature. The films were visualized with chemiluminescence, and quantitative analyses were performed by densitometry using Image-pro plus 6.0 software. The relative protein expression was compared with β-actin.

### Statistical Analysis

The data are shown as means ± SD. SPSS 16.0 software was used to analyze the significant differences among multiple groups by one-way ANOVA. *P* < 0.05 was considered to be statistically significant.

## Results

### Chromatographic Analysis of LLC

**FIGURE 1:**
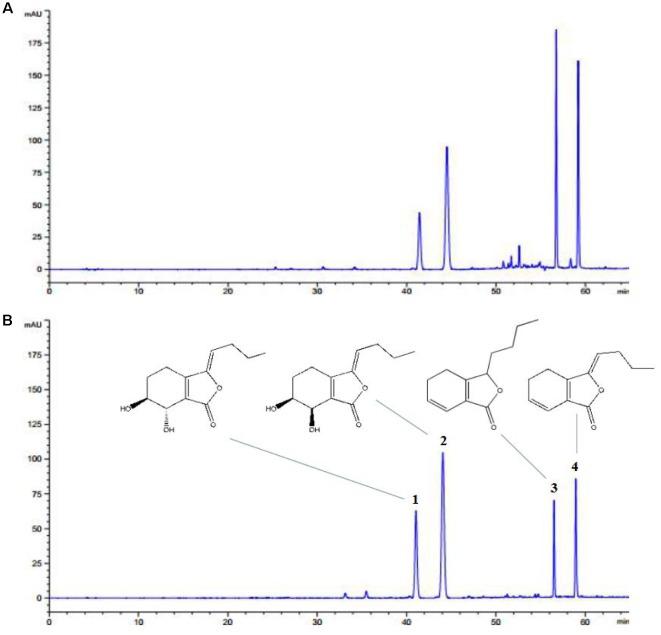
HPLC-DAD profile of LLC. Representative chromatograms of **(A)** LLC and **(B)** standard materials including (1) senkyunolide I, (2) senkyunolide H, (3) senkyunolide A and (4) Z-ligustilide.

*Ligusticum chuanxiong* is the major active component of *L. chuanxiong*, which has favorable cardioprotective effects. HPLC-DAD was performed for analysis and identification of LLC by compared with mixed standard materials. The chromatogram and structures of LLC were shown in **Figures 1A,B**. Finally, the four major compounds of LLC were identified as (1) senkyunolide I; (2) senkyunolide H; (3) senkyunolide A, and (4) Z-ligustilide. The purity of LLC was 71.56%, and the concentrations of four major compounds are as follows: (1) senkyunolide I (3.19%); (2) senkyunolide H (4.14%); (3) senkyunolide A (22.01%), and (4) Z-ligustilide (42.22%).

### LLC Ameliorates ISO-Induced Myocardial Ischemia Injury in Rat

**FIGURE 2:**
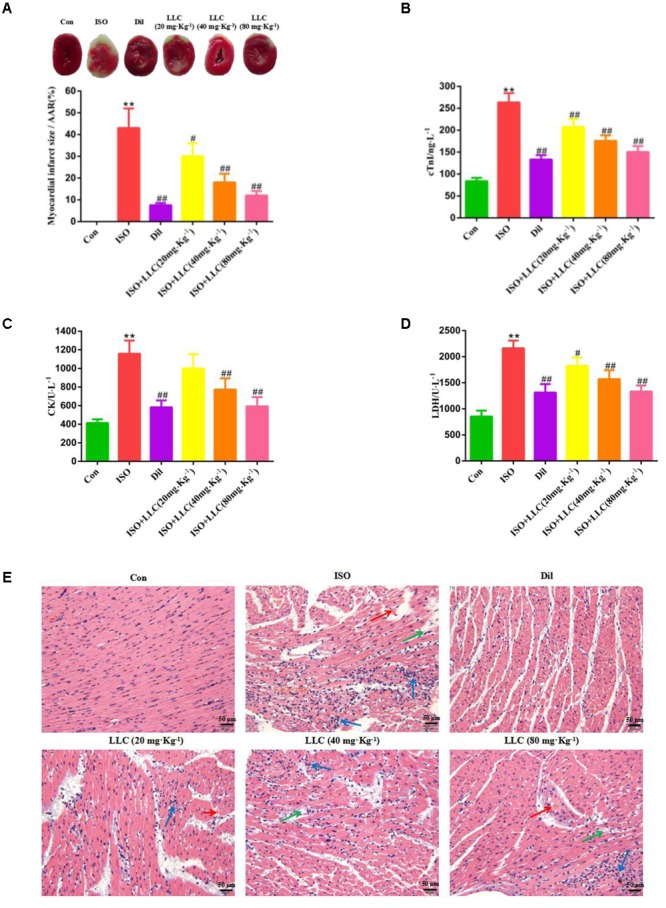
Effects of LLC on infarct size, serum biochemical indexes and histopathological changes in rats subjected to myocardial ischemia injury. **(A)** Representative images and analysis of the infarct size. The red-stained area represents viable myocardium, where the white area represents infarcted myocardium. Myocardial infarct size was expressed as percent of infarct divided by the total area at risk (AAR). **(B–D)** The serum levels of cardiac troponin (cTnI) **(B)**, creatine kinase (CK) **(C)**, and lactate dehydrogenase (LDH) **(D)** in rats are shown. **(E)** The pathological changes of myocardial ischemia injury in different groups of rats (scale bar = 50 μm). Histopathological changes are indicated by green (edema), blue (inflammatory cell infiltration ) and red (necrosis) arrows. Data were presented as means ± SD. ^∗∗^*P* < 0.01 vs. Con group, ^#^*P* < 0.05, ^##^*P* < 0.01 vs. ISO group. Con, control; ISO, isoproterenol; Dil, diltiazem.

To determine the cardioprotective effects of LLC on myocardial ischemia injury, we explored the myocardial infarct size, serum marker enzymes, production of ischemic myocardium and histopathological change. In accordance with a previous study (Shi et al., 2013), our experiment showed that the subcutaneous injection of ISO could induce a significant myocardial infarction (43.01 ± 9.45%). As shown in **Figure 2A**, the infarct size was significantly decreased in the Dil group, pretreatment of rats with LLC at doses of 20, 40, and 80 mg⋅kg^-1^ resulted in a dose-dependent decrease in infarct size when compared with ISO group.

As important indicators of myocardial injury, the activities of CK, LDH and the content of cTnI in serum were measured from experimental group rats (**Figures 2B–D**). Relative to the normal control group, the serum levels of CK, LDH, and cTnI production were dramatically increased in ISO group, while the increase was markedly suppressed by Dil and LLC from 20 to 80 mg⋅kg^-1^. There was a dose-effect relationship among the three LLC pretreated groups.

Furthermore, we also examined myocardial histopathological changes in rats pretreated with or without LLC (**Figure 2E**). Histopathology of rat hearts from control group kept normal myofibrillar structure and shape, while the ISO group revealed obvious myocardial structure disorder, myonecrosis, edema, and inflammatory cell infiltration. The pretreatment with LLC and Dil protected the cardiomyocytes from damage with the separation of myofibrils, myocardial cell swelling and diminished inflammatory cell infiltration, compared with ISO group.

Collectively, the above results demonstrated that LLC exhibits significant cardioprotective efficacy against ISO-induced myocardial ischemia injury in rats.

### LLC Improves Cell Survival in H9c2 Cardiomyocytes Under OGD

**FIGURE 3:**
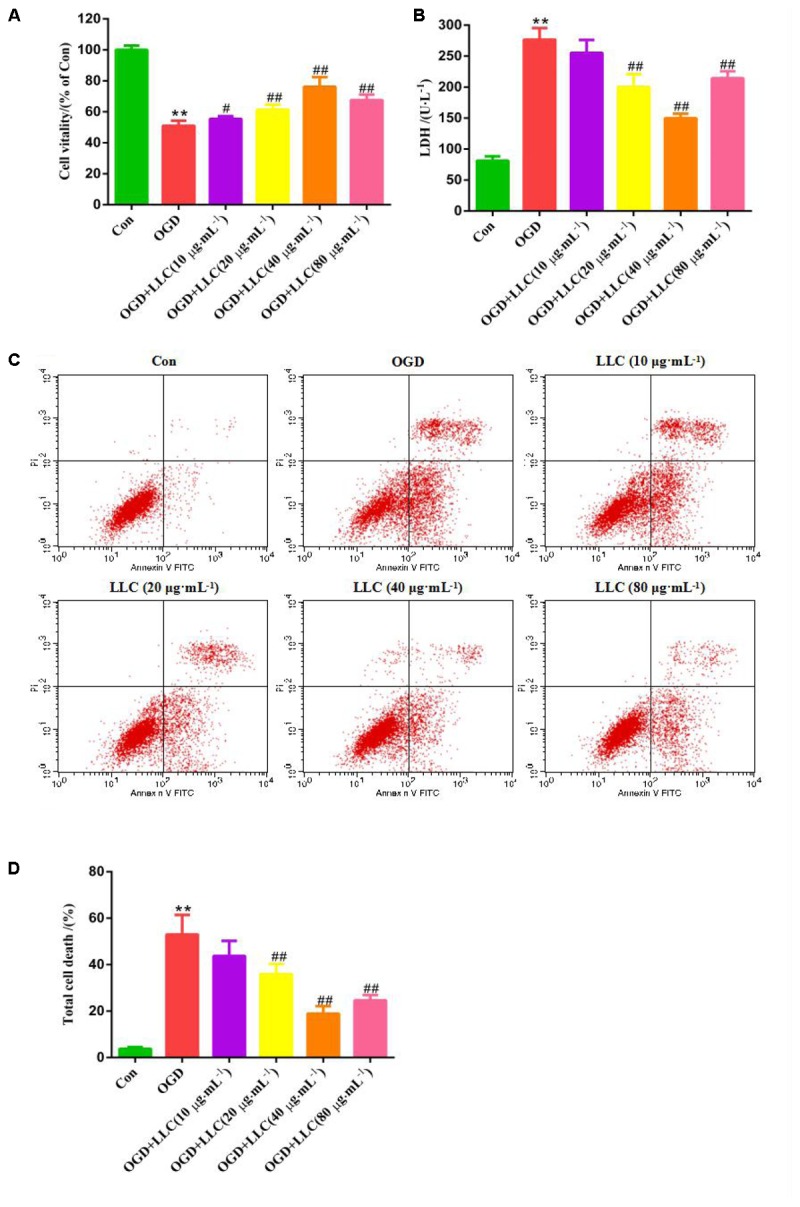
Effects of LLC on the cell viability, LDH leakage and apoptosis in H9c2 cardiomyocytes under OGD. **(A)** The cell viability was analyzed by MTT assay. **(B)** The LDH leakage in the cell culture serum was assayed. **(C)** The total cell death was observed by Annexin V-FITC/PI staining. **(D)** Quantitative assessment of total cell death rates. All experiments were repeated at least three times. Data were presented as means ± SD. ^∗∗^*P* < 0.01 vs. Con group, ^#^*P* < 0.05, ^##^*P* < 0.01 vs. OGD group. OGD, oxygen-glucose deprivation.

To determine whether LLC confers cardioprotection through its direct action on the cardiomyocytes, H9c2 cardiomyocytes were applied different concentrations of LLC and then challenged by OGD to simulate myocardial ischemia injury *in vitro*. As shown in **Figures 3A,B**, the cell viability was reduced and LDH release was increased significantly in OGD group compared with control group, which indicated that H9c2 cardiomyocytes were injured seriously under OGD. Interestingly, LLC could enhance the cell viability and decrease LDH release in a dose-dependent manner from 10 to 40 μg⋅mL^-1^ and did not have a further improvement at 80 μg⋅mL^-1^. Thus, 40 μg⋅mL^-1^ LLC was chosen for using in the pretreated group in subsequent experiments.

In addition, anti-apoptotic effects of LLC were evaluated by Annexin V-FITC/PI staining (**Figure 3C**), which reflected that control group appeared normal apoptosis. Following OGD, the total cell death rate was significantly increased, while total cell death was markedly ameliorated with LLC pretreatment (**Figure 3D**). These were further evidence that LLC could promote cell survival and reduce cell damage in H9c2 cardiomyocytes subjected to OGD.

### Effect of LLC on Autophagy Following Myocardial Ischemia Injury *in Vivo* and *in Vitro*

**FIGURE 4:**
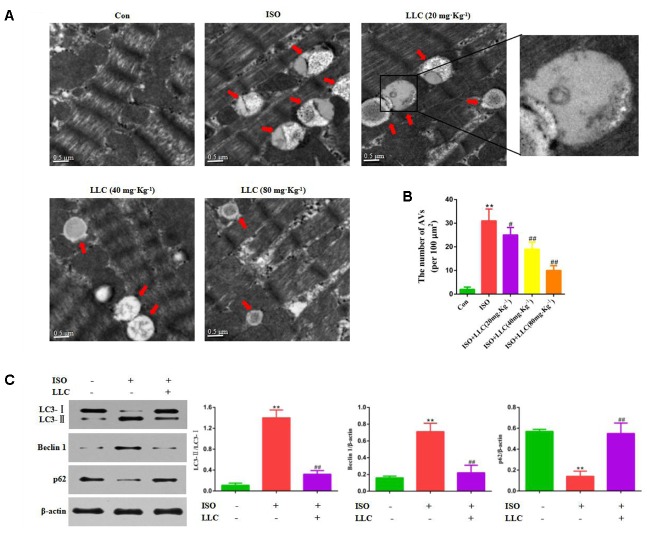
Effects of LLC on autophagy in the hearts from rats subjected to myocardial ischemia injury. **(A)** Representative transmission electron ultra-images showing autophagic vacuoles (marked with red arrows in the images, scale bar = 0.5 μm). **(B)** Quantitative analysis of the number of autophagic vacuoles in **(A)**. **(C)** Expression levels of LC3, Beclin 1 and p62 changes with LLC (80 mg⋅Kg^-1^) pretreatment. Data were presented as means ± SD. ^∗∗^*P* < 0.01 vs. Con group, ^#^*P* < 0.05, ^##^*P* < 0.01 vs. ISO group.

**FIGURE 5:**
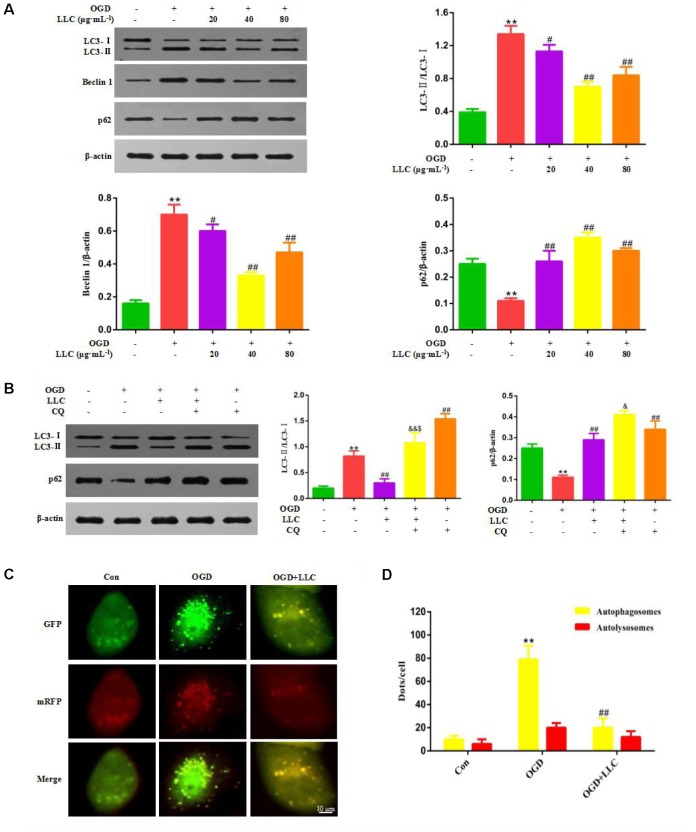
Effects of LLC on autophagy in H9c2 cardiomyocytes under OGD. **(A)** Expression levels of LC3, Beclin 1 and p62 changes with LLC pretreatment. **(B)** Expression levels of LC3 and p62 changes in the presence or absence of 5 μmol⋅L^-1^ chloroquine and 40 μg⋅mL^-1^ LLC. **(C)** H9c2 cardiomyocytes were transfected with mRFP-GFP-LC3 and observed by fluorescent microscope (scale bar = 10 μm). **(D)** Mean number of autophagosomes represented by yellow dots in merged images and autolysosomes represented by red dots in merged images per cell. All experiments were repeated at least three times. Data were presented as means ± SD. ^∗∗^*P* < 0.01 vs. Con group, ^##^*P* < 0.01 vs. OGD group, ^&^*P* < 0.05, ^&&^*P* < 0.01 vs. OGD+LLC group, ^$^*P* < 0.05 vs. OGD+CQ group. CQ, chloroquine.

Next, whether and how autophagy plays a role in LLC-mediated cardioprotection were investigated. Firstly, we directly observed the induction of autophagy in cardiomyocytes of myocardial ischemia rats by transmission electron microscopy. Numerous accumulation of autophagic vacuoles (AVs) characterized by one limiting membrane or double membrane containing degraded cytoplasmic contents was found in ISO group (**Figures 4A,B**). After pretreatment with LLC, the formation of AVs was significantly decreased compared to ISO group. Then we examined the expression levels of autophagic markers in myocardial ischemia hearts (**Figure 4C**) and H9c2 cardiomyocytes (**Figure 5A**), such as LC3-II/LC3-I ratio, Beclin 1 and p62. The expressions of Beclin 1 and LC3-II/LC3-I ratio were upregulated, and the expression of p62 was downregulated in rat hearts and H9c2 cardiomyocytes subjected to myocardial ischemia injury when compared to control group, but it was markedly reversed by pretreatment of LLC. These results suggested that LLC protected the heart from myocardial ischemia injury by inhibiting autophagy.

### LLC Recovers Autophagic Flux in H9c2 Cardiomyocytes Exposed to OGD

In order to clarify the effect of LLC on actual autophagic flux, chloroquine (CQ) was administered with LLC before OGD. CQ acts as a potent autophagy inhibitor at the late stage of autophagic flux, which prevents autophagosome-lysosome fusion and subsequently inhibits degradation of proteins via increase of the lysosomal pH. Autophagic flux was quantified as differences in LC3-II/LC3-I ratio obtained in the presence and absence of CQ. The results of western blot indicated that CQ significantly elevated LC3-II/LC3-I ratio and the expression of p62 compared with OGD group, which implied that CQ blocked OGD-accelerated autophagic flux by inhibiting autophagosome clearance (**Figure 5B**). Likewise, LLC combined with CQ reduced LC3-II/LC3-I ratio compared with CQ alone (**Figure 5B**), which indicated that the inhibition of autophagy by LLC occurred in the upstream of autophagic flux.

We further confirmed whether LLC recovered OGD-accelerated autophagic flux by suppression of autophagosome formation using mRFP-GFP-LC3 assay. As shown in **Figures 5C,D**, in OGD group, a significantly increased yellow fluorescent dots were observed, which suggested that autophagosome formation was enhanced and autophagic flux was accelerated after OGD. In LLC-pretreated group, yellow fluorescent dots were markedly reduced, and less red yellow fluorescent dots were observed, which indicated that LLC might suppress autophagosome formation. Collectively, these data demonstrated that OGD-induced autophagic flux was restored by LLC.

### PI3K/Akt/mTOR Signaling Pathway Is Involved in the Cardioprotective Effects of LLC

**FIGURE 6:**
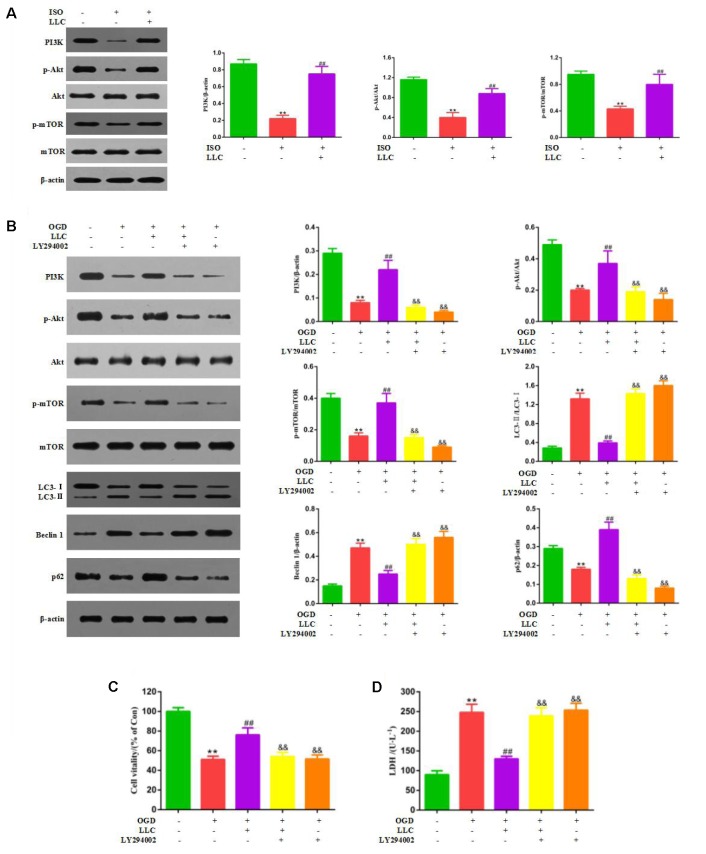
PI3K/Akt/mTOR pathway is involved in the cardioprotective effects of LLC. **(A)** Expression levels of PI3K, p-Akt, Akt, p-mTOR and mTOR in hearts were analyzed. **(B)** Representative western blots of PI3K (p110α), p-Akt (Ser473), Akt, p-mTOR (Ser2448), mTOR, LC3, Beclin 1 and p62 in the presence or absence of 20 μmol⋅L^-1^ LY294002 and 40 μg⋅mL^-1^ LLC. **(C)** The cell viability was analyzed by MTT assay. **(D)** The cell injury was detected by LDH measurements. All experiments were repeated at least three times. Data were presented as means ± SD. ^∗∗^*P* < 0.01 vs. Con group, ^##^*P* < 0.01 vs. OGD group, ^&^*P* < 0.05, ^&&^*P* < 0.01 vs. OGD+LLC group.

**FIGURE 7:**
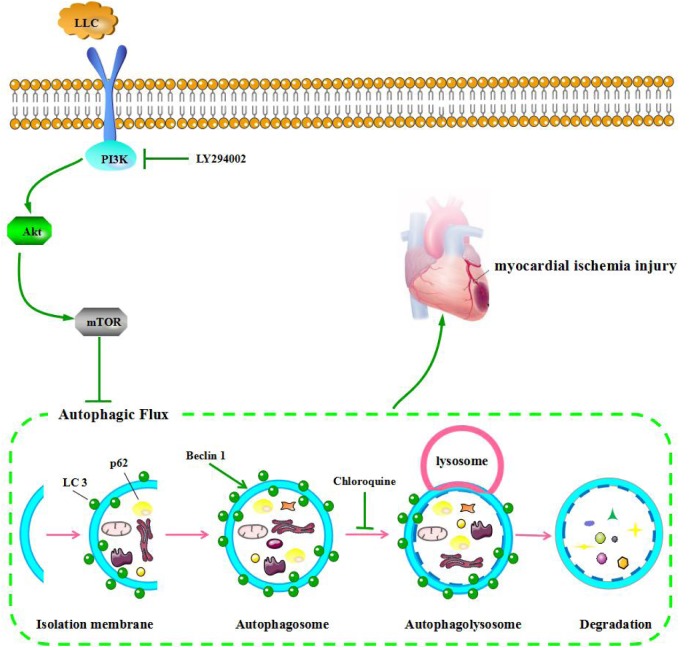
LLC ameliorates myocardial ischemia injury through inhibiting autophagy by the activation of the PI3K/Akt/mTOR pathway.

Next, we set out to explore the role of the PI3K/Akt/mTOR signaling pathway in the protective effects of LLC against myocardial ischemia injury. Western blot analysis indicated that the expressions of PI3K (p110α), p-Akt (Ser473), and p-mTOR (Ser2448) were markedly decreased in ISO group compared with control group, but it was remarkably enhanced by LLC (**Figure 6A**), similar results were observed in cardiomyocytes subjected to OGD (**Figure 6B**). To further confirm the relationship between PI3K/Akt/mTOR signaling pathway and LLC-mediated cardioprotection, a specific PI3K inhibitor LY294002 was applied in this study. The treatment of LY294002 could block LLC-upregulated PI3K, p-Akt and p-mTOR. It also abrogated LLC-induced downregulation of LC3-II/LC3-I ratio and Beclin 1 level as well as upregulation of p62 level during OGD (**Figure 6B**). In addition, LY294002 abolished LLC-improved cell viability (**Figure 6C**) and LDH release (**Figure 6D**) in H9c2 cardiomyocytes subjected to OGD. Taken together, these results provided strong evidence that LLC conferred cardioprotective effects in part by inhibiting autophagy dysfunction through the activation of PI3K/Akt/mTOR signaling pathway (**Figure 7**).

## Discussion

Cardiovascular diseases still remain a leading cause of morbidity and mortality in the worldwide, and the ischemic heart disease occupies a primary place in the cause of cardiovascular diseases, especially for acute myocardial infarction (Mozaffarian et al., 2015). Although modern medicine has made great progress in improving blood supply and relieving angina, there are still few effective measures to prevent occurrence and development of myocardial ischemia injury. However, in the current research of ischemic heart disease treatment, emerging active components from traditional Chinese medicine have been regarded as the focus in pharmaceutical development.

Lactone component from *L. chuanxiong* (LLC) represents the major active component isolated from the traditional Chinese herb *L. chuanxiong* Hort., which has been used as a medicinal plant to treat cardiovascular diseases in China for thousands of years. In the present study, we found that LLC concentration -dependently ameliorated myocardial ischemia injury in rats, as manifested by a reduction of the infarct size, a decrease in the serum levels of cardiac enzymes and an improvement of pathological changes. To further confirm the speculation, we then sought to elucidate the effect of LLC on myocardial cells, and H9c2 cardiomyocytes were challenged by OGD to mimic ischemia *in vitro*. The protection is related to the direct action of LLC on the cardiomyocytes as LLC has similar improvement on the cell survival in cardiomyocytes subjected to OGD. These results are in accordance with some previous reports (Liang et al., 2004), which showed a cardioprotective effect of LLC against myocardial ischemia injury *in vitro* and *in vivo*. Additionally, we demonstrated that the role of LLC in myocardial ischemia injury was related to the inhibition of autophagy.

Autophagy plays a pivotal role in myocardial ischemia injury. A growing number of studies in recent years have illustrated that autophagy is involved in the pathological process in myocardial ischemia injury, when the organism is subjected to starvation, ischemia, hypoxia, ATP depletion, endoplasmic reticulum stress and reactive oxygen species (Yan et al., 2013). Nevertheless, the functional role of autophagy in cell survival and death pathways associated with heart damage has not yet been fully clarified. Several lines of evidence suggest that autophagy may improve cell survival by clearing damaged proteins and aging organelles to generate the intracellular building blocks which were required to maintain vital structure and function during nutrient-limiting condition (Kuma et al., 2004; Matsui et al., 2007). Other lines of evidence suggest autophagy may also promote cell death through excessive self-digestion and degradation of essential cellular constituents (Hamacher-Brady et al., 2007), indicating autophagy is a double-edged sword in myocardial ischemia injury. In this study, we confirmed that autophagy was initiated with a significant increase in autophagic vacuoles, enhancement of LC3-II/LC3-I ratio and Beclin 1 level and decrease in p62 level in the myocardial ischemia injured rats, LLC could counteract the autophagy process as evidence by a decreased autophagic vacuoles, LC3-II/LC3-I ratio and Beclin 1 level as well as an elevation of p62 level. And this is consistent with the effects of LLC on the expressions of autophagic markers in H9c2 cardiomyocytes subjected to OGD. Collectively, our data indicated that autophagy had exceeded its capacity to protect cardiomyocytes against myocardial ischemia injury, which is a detrimental role in ischemic myocardium, while the modulation of autophagy by LLC is cardioprotective. However, some studies showed that the induction of autophagy is a beneficial role in ischemia phase (Xiao et al., 2015). The contradictory results may be interpreted by the different cell types, animal species, modeling methods and degree of injury. And some researchers have proposed that the role of autophagy in myocardial ischemia injury depends on the extent of autophagy. Moderate autophagy is considered to be beneficial for cardiomyocytes survival but excessive autophagy aggravates cardiomyocytes death (Xie et al., 2012; Lavandero et al., 2013). So it is very important to evaluate the level of autophagy quickly and accurately for recognizing biological functions of autophagy in myocardial ischemia injury.

Autophagic flux is a consecutive and dynamic process involving in nucleation, autophagosome formation, fusion of autophagosomes to lysosomes, and degradation in lysosomes, and through its detection can scientifically and comprehensively reflect the level of autophagy. Some previous researches partial to evaluate the extent of autophagy by the assessment of autophagosome formation at a single time point or observe autophagic phenomenon, while the results could be misleading because the number of autophagosomes often dissociates from the level of autophagy (Hariharan et al., 2011). For example, the accumulation of autophagosomes results from a block in trafficking to lysosomes, whereas an increase in autophagic vacuoles may just reflect a reduction in degradative activity and cannot indicate the upregulation of the level of autophagy. In this study, lysosomal inhibitor CQ was applied as the major tool for evaluating the effects of LLC pretreatment on the actual autophagic flux *in vitro*. In agreement with a previous study (Zhong et al., 2017), CQ markedly increased LC3-II/LC3-I ratio and p62 level, which suggested that late phase of autophagic flux was blocked by CQ. Coadministration of LLC and CQ reduced LC3-II/LC3-I ratio as compared with CQ alone, indicating that LLC may recover OGD-accelerated autophagic flux by inhibiting the upstream of autophagic flux. To further elucidate the effects of LLC pretreatment on autophagosome formation during OGD, autophagic flux was monitored by mRFP-GFP-LC3 assay, we found that LLC restore autophagic flux via suppressing autophagosome formation.

PI3K/Akt pathway is a classical signaling pathway that plays a key role in normal cellular functioning, including proliferation, adhesion, migration, invasion, energy metabolism, protein synthesis, and prosurvival. The kinase mammalian target of rapamycin (mTOR) is a key regulator of autophagy, the activity of which is enhanced by factors that activate the PI3K/Akt pathway. Previous reports indicated that PI3K/Akt pathway was activated by the major compounds of LLC and mediated protection against OGD-induced injury (Qi et al., 2012; Xu et al., 2016). Therefore, to further explore the underlying mechanisms of LLC-induced cardioprotection, we targeted the PI3K/Akt/mTOR signaling pathway. It was found that LLC pretreatment significantly increased the expression of PI3K and phosphorylation of Akt and mTOR in rats and H9c2 cardiomyocytes. Meanwhile, as a specific PI3K inhibitor, LY294002 abolished the improvement of LLC on OGD-altered autophagy dysfunction and cardiomyocyte survival. Thus, we can infer that the PI3K/Akt/mTOR pathway is involved in the modulation of cardioprotection of LLC in myocardial ischemia injury.

## Conclusion

The pretreatment of LLC significantly improved cardiomyocyte survival, and recovered the heart damage after myocardial ischemia injury in rats. Such cardioprotection is at least partially mediated by restoration of autophagic flux through the activation of the PI3K/Akt/mTOR signaling pathway. These findings reveal that LLC may be a potential therapeutic strategy in the protection of myocardium from ischemia disease.

## Author Contributions

LF and XJ designed the research study. GW, GD, JS, MZ, YL, XH, and ZK performed the experiments. YZ, HQ, FW, and NJ analyzed the data. GW wrote the manuscript. All authors read and approved the final manuscript.

## Conflict of Interest Statement

The authors declare that the research was conducted in the absence of any commercial or financial relationships that could be construed as a potential conflict of interest.
